# Preoperative Ultrasonography in the Evaluation of Suspected Familial Non-Medullary Thyroid Cancer: Are We Able to Predict Multifocality and Extrathyroidal Extension?

**DOI:** 10.3390/jcm10225277

**Published:** 2021-11-13

**Authors:** Giorgio Grani, Gianluca Cera, Giovanni Conzo, Valeria Del Gatto, Cira Rosaria Tiziana di Gioia, Marianna Maranghi, Piernatale Lucia, Vito Cantisani, Alessio Metere, Rossella Melcarne, Maria Carola Borcea, Chiara Scorziello, Rosa Menditto, Marco Summa, Marco Biffoni, Cosimo Durante, Laura Giacomelli

**Affiliations:** 1 Department of Translational and Precision Medicine, Sapienza University of Rome, Viale del Policlinico, 155, I-00161 Rome, Italy; giorgio.grani@uniroma1.it (G.G.); Cera.1637917@studenti.uniroma1.it (G.C.); valeria.delgatto@uniroma1.it (V.D.G.); marianna.maranghi@uniroma1.it (M.M.); piernatale.lucia@uniroma1.it (P.L.); 2 Department of Cardiothoracic Sciences, University of Campania Luigi Vanvitelli, I-80131 Naples, Italy; giovanni.conzo@unicampania.it; 3 Department of Radiological, Oncological and Pathological Sciences, Sapienza University of Rome, Viale del Policlinico, 155, I-00161 Rome, Italy; cira.digioia@uniroma1.it (C.R.T.d.G.); vito.cantisani@uniroma1.it (V.C.); 4 Department of Surgical Sciences, Sapienza University of Rome, Viale del Policlinico, 155, I-00161 Rome, Italy; alessio.metere@uniroma1.it (A.M.); rossella.melcarne@uniroma1.it (R.M.); mariacarola.borcea@uniroma1.it (M.C.B.); chiara.scorziello@uniroma1.it (C.S.); rosa.menditto@uniroma1.it (R.M.); marco.summa@uniroma1.it (M.S.); marco.biffoni@uniroma1.it (M.B.); laura.giacomelli@uniroma1.it (L.G.)

**Keywords:** TIRADS, thyroid cancer, thyroid nodules, multifocality

## Abstract

Family history of thyroid cancer increases the risk of harboring thyroid malignancies that end up having extrathyroidal extension (ETE) and multifocality on histology; some authors suggest a more aggressive surgical approach. Their pre-operative identification could allow more conservative surgical procedures if none of these features are suspected. Our aim was to assess if neck ultrasonography could identify or exclude multifocality or ETE in these patients to tailor the extent of surgery. This retrospective study included patients with previous thyroid surgery, ≥1 first-grade relative with thyroid cancer, and who had undergone pre-surgical ultrasound. ETE was suspected in the case of thyroid border interruption or gross invasion of perithyroidal tissues. Multiple suspicious nodules were defined as suspicion of multifocal cancer. The cohort consisted of 45 patients (median age 49 years, 40 with thyroid cancer, 30 females). The positive predictive value of ultrasonography in predicting multifocality and ETE was 57.14% (25.25–84.03) and 41.67% (21.5–65.1%), respectively, while the negative predictive values were 63.2% (56.4–69.4%) and 72.7% (63.3–80.5%). Pre-operative ultrasound examination is unable to reliably identify or exclude multifocal disease or extrathyroidal extension. In patients scheduled for surgery and with a first-degree relative affected by DTC, a “negative” pre-operative US report does not exclude the potential finding of multifocality and ETE at final histopathology.

## 1. Introduction

Differentiated thyroid cancer (DTC) is commonly associated with a good prognosis and excellent survival rates [1]. Treatment strategies for DTC patients are increasingly tailored to individual patients’ needs to guarantee that the benefits of more aggressive (or conservative) therapies outweigh the risks of adverse outcomes. This approach is advocated by international guidelines [2,3], with some issues still debated [4]. Given the low risk of death from DTC, treatment plans mostly rely on estimates of patients’ risk of persistence or recurrence of disease, which guides decisions on surgery (e.g., lobectomy vs. total thyroidectomy vs. total thyroidectomy with prophylactic central neck dissection) and post-surgical treatment (e.g., radioiodine administration). Many tumor characteristics, including size, multifocality, extra-thyroidal extension (ETE), vascular invasion, incomplete surgical resection, lymph node involvement, and metastasis, have been associated with an increased risk of recurrence, warranting a more aggressive surgical approach and/or radioiodine treatment [2,3].

A family history of DTC is associated with some of these higher-risk features. Familial cases are characterized by younger age at presentation, increased risk of recurrence, and potentially more aggressive disease, with tumors showing multifocality and extra-thyroidal extension (ETE) more frequently than sporadic cases. However, the clinical implications of these findings on tumor screening, treatment, and prognosis remain controversial [5,6,7]. In total, 5–15% of DTC cases can be defined as familial in the context of multiple tumor syndromes (such as FAP, Gardner, Peutz–Jeghers syndromes, and Carney complex), or as familial non-medullary thyroid cancers (FNMTC) when at least two or three first-degree relatives are affected by DTC [6,8]. Having only one first-degree relative affected by DTC does not satisfy FNMTC criteria, but it has indeed been associated with some of its clinical features, including the histological presence of multifocality and ETE [5]. This may be partially explained by the fact that at least some of these cases are indeed familial forms that have not met diagnostic criteria yet [6].

Since familiarity increases the risk of both ETE and multifocality, more aggressive therapeutic approaches are advocated by default by some authors to prevent recurrences in patients with a family history of DTC. Nevertheless, a reliable preoperative assessment of ETE and multifocality may allow clinicians to propose a more conservative surgical approach (i.e., lobectomy) if none of these features are suspected. On the other hand, the identification of ETE and multifocality can favor total thyroidectomy and reduce the risk of having to perform a completion thyroidectomy [9]. Thyroid ultrasound (US) can be used to this scope, as it is the most informative imaging technique in evaluating number, size, location and characteristics of both thyroid nodules [10] and loco-regional lymph nodes [11]. US predictive power in identifying ETE when compared to post-surgical histopathological results is variable and suboptimal, and relevant interobserver variability has been reported; even so, neck US could be useful especially as a rule-out test given the higher negative predictive value reported in several studies [12,13].

In this study, we aimed to assess if preoperative neck US can identify or exclude multifocality and/or ETE in patients scheduled for surgery for a suspicious thyroid nodule and with family history of DTC, to clarify whether such history represents an “unpredictable” risk factor that warrants broader surgery, or if it is associated with detectable features allowing for tailored conservative treatment.

## 2. Materials and Methods

We carried out a retrospective observational study at the Thyroid Disease Clinic in Sapienza University of Rome. All patients met the following inclusion criteria: (a) previous thyroid surgery for sonographically and/or cytologically suspicious thyroid nodules; (b) complete histopathological report; (c) at least one first-grade relative affected by DTC (as known at the time of surgery); and (d) at least one pre-surgical neck ultrasound with available images and/or report, carried out to evaluate suspicious thyroid nodules, to perform FNA procedures, or as pre-surgical mapping. All patients were retained in the final analysis regardless of the final histological diagnosis. Ultrasound images and reports were examined by an experienced clinician (G.G.) to assess the suspicion of extra-thyroidal extension and/or multifocal disease between November 2015 and December 2020. While this specific analysis was retrospectively performed, the reporting and collection of sonographic features was part of a prospective, pre-specified study; sonographic examinations were performed at the Thyroid Disease Clinic following a standard ultrasound scanning protocol. Specifically, extra-thyroidal extension was suspected in the presence of images suggestive of thyroid border interruption or of nodular invasion of perithyroidal tissues. Suspicion of multifocal disease was defined if multiple suspicious nodules were visible. American College of Radiology (ACR) Thyroid Imaging Reporting and Data System (TIRADS) scores were determined for each patient in order to estimate their malignancy risk.

Histopathological reports were examined to identify the description of microscopic or macroscopic extra-thyroidal extension and unilateral (including lesions in one lobe and isthmus) or bilateral multifocal disease.

US performance was assessed using the final histology report as the reference standard, using Fisher’s exact test to determine the statistical significance of US findings, and estimating sensitivity, specificity, positive and negative LR, and accuracy, each with 95% CI. Statistical analysis was performed with Microsoft Excel and MedCalc Software.

The study was conducted according to the guidelines of the Declaration of Helsinki and approved by the Ethics Committee of Sapienza University of Rome (protocol code 4233, 12 December 2016). Informed consent was obtained from all subjects involved in the study.

## 3. Results

The final cohort consisted of 45 patients with a family history of thyroid cancer and complete sonographic data, aged 24 to 79 (median 49; IQR 3–63), mostly female (67%). Overall, 5 patients belonged to kindreds with 1 family member affected by DTC, 30 patients to kindreds with 2 DTCs, and 10 patients to kindreds with 3 or more DTCs. Relevant clinical features of this cohort are summarized in Table 1.

Thirty-seven patients had undergone FNA of suspicious nodules, classified according to the Italian Thyroid Cytology Classification System [14] as TIR2 to TIR5 (with indeterminate TIR3 class divided in a lower-risk group, TIR3A, and a higher-risk group, TIR3B), which approximately correspond to Bethesda [15] classes II to VI. One other patient had a FNA result of TIR1 (non-diagnostic). Seven patients had no FNA reports (not performed or not available). 

### 3.1. Ultrasound and Surgical Pathology Results

US neck images and reports review showed that 25 (56%) patients had bilateral nodules, and 11 (24%) had unilateral multinodular disease; 9 (20%) patients had a solitary nodule. Of the 25 patients with bilateral nodules, 7 (16%) had a suspicion of bilateral multifocal DTC because of the presence of at least two suspicious nodules in the two lobes. The remaining 18 patients with bilateral nodules and the 11 patients with unilateral nodules had only one suspicious lesion, therefore having no suspicion of multifocality. Disruption of the thyroid capsule or a clear invasion of perithyroidal tissue was observed in 12 patients (27%) (Table 1).

Histological examination revealed 5 cases of benign adenomas and 40 cases of thyroid cancer, mostly papillary thyroid cancer. The median diameter of malignant nodules was 8 mm (IQR 5–13.5). A total of 18 patients (40%) had bilateral (*n* = 13) or unilateral (*n* = 5) multifocal disease. Microscopic ETE was observed in 13 cases (29%), while macroscopic ETE was observed in just one case (Table 1).

### 3.2. Diagnostic Performance of US Examination

The crosstabulations of US and surgical pathology reports for multifocality and extrathyroidal extension are illustrated in Table 2 and Table 3, respectively. Among the 7 patients with US suspicion of multifocality, 4 (57%) had multifocal disease according to pathology reports. 14 (37%) out of 38 patients without multiple suspicious nodules had multifocal disease anyway. ETE, on the other hand, was observed in 5 cases (42%) out of 12 suspicious US figures, and in 9 (27%) cases out of 33 without such appearances.

Table 4 summarizes the diagnostic performance of US findings in predicting multifocality and ETE. Even though absolute frequencies of confirmed ETE and multifocality seem to be higher in patients with US suspicion of these characteristics, the performance is not statistically significant, nor clinically useful. This is reflected in the non-significant results of positive and negative likelihood ratios.

We then evaluated whether more suspicious nodules, based on the ACR TIRADS classification, were more likely to have ETE or multifocal disease. Only nodules classified as TR5 have a higher rate of ETE at histology (Table 5).

## 4. Discussion

In recent years, the clinical guideline recommendations were revised in order to avoid over-diagnosis in patients with low-risk thyroid nodules, aiming to promptly identifying patients with advanced or high-risk tumors requiring aggressive treatment approaches [16]. This involved discouraging screening programs [17,18], biopsy of suspicious subcentimeter nodules [10], and favoring less extensive surgery [19,20] and radioiodine use [21], as well as reducing the burden of post-surgical follow-up examinations [22,23]. These efforts are consistent with the general trend in reducing low-value care [24]. Some of these recommendations do not apply to patients with one or more first-degree relatives with a history of thyroid cancer due to the potentially more aggressive nature of their neoplasms [6,25]. According to some reports, patients with first-degree relatives affected by DTC have an increased risk of multifocality and extrathyroidal extension: these findings, according to some authors, would justify a more extensive surgery [26]. Actually, when thyroid surgery is advocated for a suspected familial thyroid cancer, a number of consensus statements report data to favor total thyroidectomy (e.g., Japan Association of Endocrine Surgery [19], and the American Association of Endocrine Surgeons documents [27]). The debate on whether familiarity can be counted as a risk factor in DTC patients is still ongoing, with some studies reporting more aggressive behavior and a higher rate of recurrence [28,29,30], and other studies finding no differences [31]. Still, the impact of microscopic ETE on disease recurrence is not clear, as some studies reported no difference between patients with and without microscopic ETE [32], while a recent meta-analysis documented an increased risk (with no effect on survival) [33].

In this study, we evaluated whether pre-operative US examination of patients eligible for surgery for a suspicious thyroid nodule and a family history of thyroid cancer was able to identify features suggestive of multifocality and microscopic extrathyroidal extension in order to potentially restrict total thyroidectomy to individuals in which one of these situations was detected or suspected. We have found that ultrasonography was not able to reliably detect microscopic extrathyroidal involvement or multifocal disease, as reported by the surgical pathology report. These results are consistent with most of the studies conducted on not selected DTC cohorts [12,13,34]; in some cases, with sufficient NPV to rule out ETE [35]. Some authors reported better performance of neck US in detecting thyroid cancer minimal extrathyroidal extension (with a NPV of 76.2% and a PPV of 81.4%) [36]. The latter study, however, evaluated larger tumors (1.81 ± 0.61 cm in patients with ETE), and the matching of the nodule identified by sonography and histopathology was not guaranteed. Another possible explanation of this discrepancy is that in our cohort, we retained nodules that were confirmed to be benign, but were sonographically assessed as potentially malignant. This figure dilutes the number of nodules that may actually have an extrathyroid extension at final histology. Not surprisingly, we have only found that more suspicious nodules (classified as ACR TIRADS 5) are more likely to present with ETE: it was reported that irregular margins increase the risk of completion thyroidectomy [37].

The present study has some limitations. First of all, the sample size was quite small, and more insight may be derived by the study of larger cohorts; for the same reason, we were unable to stratify our cohort according to the number of affected relatives (FNMTC is usually defined as the occurrence of the disease in two or more first-degree relatives of the patient). Furthermore, in the case of multifocal cancer, the size of the non-dominant foci is not available in many cases, not allowing for a stratification of very small foci (<1 mm) and larger foci, potentially assessable by sonography. 

It remains to be elucidated whether ETE or microscopic involvement of the same or contralateral lobe affects the short and long-term outcomes of DTC patients and justifies a more aggressive surgical approach: their impact in the context of a familial DTC occurrence is still uncertain [6,38].

## 5. Conclusions

In patients with one or more first-degree relatives with DTC scheduled for surgery for suspicion of thyroid malignancy, pre-operative ultrasound examination is unable to reliably identify or exclude multifocal disease or extrathyroidal extension. Thus, the extent of the surgical approach cannot be reduced by a “negative” US report if clinicians and patients are worried about the potential subsequent pathological findings of multifocality and ETE. 

## Figures and Tables

**Table 1:** Clinical, sonographic, and pathological features of the study cohort.

Clinical Features	n. or Median	% or IQR
Age (years)	49	39–63
Gender, female	30	67
Gender, male	15	33
**Pre-Surgical Cytology ^1^:**		
Not available/nondiagnostic (category Tir 1)	8	18
Benign (category Tir 2) *	4	9
Indeterminate (categories Tir 3A and 3B)	15	33
Suspicious (category Tir 4)	9	20
Malignant (category Tir 5)	9	20
**Sonographic Features**	**n.**	**%**
Single nodule	9	20
Multiple nodules, unilateral	11	24
Multiple nodules, bilateral	25	56
**Suspicion of:**		
Multiple malignant foci, unilateral	0	0
Multiple malignant foci, bilateral	7	16
Extra-thyroidal extension	12	27
**Histology Report**	**n. or median**	**% or IQR**
Tumor size (mm) *	8	5–13.5
PTC, classic variant	23	51
PTC, follicular variant	10	22
PTC, solid variant	1	2
PTC, variant not specified	3	7
FTC, minimally invasive	2	4
Anaplastic thyroid cancer	1	2
Benign *	5	11
Multiple malignant foci, unilateral	5	11
Multiple malignant foci, bilateral	13	29
ETE, microscopic	13	29
ETE, macroscopic	1	2
Total	45	100

^1^ Results according to the Italian Thyroid Cytology Classification System. Abbreviations: ETE: extrathyroidal extension; FNAC: fine-needle aspiration cytology; FTC: follicular thyroid cancer; PTC: papillary thyroid cancer. * Small foci of thyroid cancer were reported in this study even when the nodule submitted to cytology was shown to be benign, given the specific aim of the study. Patients with final benign histology were retained in the analysis because the whole cohort was submitted to surgery for concerns of malignancy (cytological or sonographical suspicion, in the context of family history of thyroid cancer) and the pre-surgical sonographic evaluation was performed for suspected cancer.

**Table 2:** Multifocality according to US and histopathological reports.

US Features	Histopathological Features		Total
	Multifocality	No multifocality	
Multifocality	4 (57%)	3 (43%)	7
No multifocality	14 (37%)	24 (63%)	38
Total	18	27	45

**Table 3:** Extrathyroidal extension according to US and histopathological reports.

US Features	Histopathological Features		Total
	ETE	No ETE	
ETE	5 (42%)	7 (58%)	12
No ETE	9 (27%)	24 (73%)	33
Total	14	31	45

**Table 4:** Diagnostic performance of US findings in predicting multifocality and ETE.

	Multifocality		ETE	
	value	95% CI	value	95% CI
Sensitivity	22.2%	6.41% to 47.64%	35.71%	12.76% to 64.86%
Specificity	88.89%	70.84% to 97.65%	77.42%	58.90% to 90.41%
Positive LR	2.00	0.51 to 7.89	1.58	0.61 to 4.12
Negative LR	0.88	0.66 to 1.16	0.83	0.54 to 1.28
PPV	57.14%	25.25% to 84.03%	41.67%	21.50% to 65.07%
NPV	63.16%	56.42% to 69.42%	72.73%	63.33% to 80.46%
Accuracy	62.22%	46.54% to 76.23%	64.44%	48.78% to 78.13%

ETE: extra-thyroidal extension; LR: likelihood ratio; PPV: positive predictive value; NPV: negative predictive value.

**Table 5:** Extrathyroidal extension and multifocality according to ACR TIRADS classification.

		ETE		Multifocality		Total
		No	Yes	No	Yes	
ACR TIRADS	TR2	4 (100%)	0 (0%)	4 (100%)	0 (0%)	4
	TR3	2 (100%)	0 (0%)	2 (100%)	0 (0%)	2
	TR4	13 (81%)	3 (19%)	10 (63%)	6 (37%)	16
	TR5	12 (52%)	11 (48%)	11 (48%)	12 (52%)	23
p		0.077		0.141		
TR2 to TR4		19 (86%)	3 (14%)	16 (73%)	6 (27%)	22
TR5		12 (52%)	11 (48%)	11 (48%)	12 (52%)	23
p		0.023		0.130		
Total		31 (69%)	14 (31%)	27 (60%)	18 (40%)	45

ACR: American College of Radiology; ETE: extra-thyroidal extension; TIRADS: Thyroid Imaging Reporting and Data System.

## Data Availability

The data presented in this study are available on request from the corresponding author. The data are not publicly available due to patients’ data confidentiality.

## References

[1] Links T.P. (2005). Life Expectancy in Differentiated Thyroid Cancer: A Novel Approach to Survival Analysis. Endocr. Relat. Cancer.

[2] Haugen B.R., Alexander E.K., Bible K.C., Doherty G.M., Mandel S.J., Nikiforov Y.E., Pacini F., Randolph G.W., Sawka A.M., Schlumberger M. (2016). 2015 American Thyroid Association Management Guidelines for Adult Patients with Thyroid Nodules and Differentiated Thyroid Cancer: The American Thyroid Association Guidelines Task Force on Thyroid Nodules and Differentiated Thyroid Cancer. Thyroid.

[3] Filetti S., Durante C., Hartl D., Leboulleux S., Locati L.D., Newbold K., Papotti M.G., Berruti A., ESMO Guidelines Committee (2019). Thyroid Cancer: ESMO Clinical Practice Guidelines for Diagnosis, Treatment and Follow-Up. Ann. Oncol.

[4] Luster M., Aktolun C., Amendoeira I., Barczyński M., Bible K.C., Duntas L.H., Elisei R., Handkiewicz-Junak D., Hoffmann M., Jarzab B. (2019). European Perspective on 2015 American Thyroid Association Management Guidelines for Adult Patients with Thyroid Nodules and Differentiated Thyroid Cancer: Proceedings of an Interactive International Symposium. Thyroid.

[5] Mazeh H., Benavidez J., Poehls J.L., Youngwirth L., Chen H., Sippel R.S. (2012). In Patients with Thyroid Cancer of Follicular Cell Origin, a Family History of Nonmedullary Thyroid Cancer in One First-Degree Relative Is Associated with More Aggressive Disease. Thyroid.

[6] Capezzone M., Robenshtok E., Cantara S., Castagna M.G. (2021). Familial Non-Medullary Thyroid Cancer: A Critical Review. J. Endocrinol. Investig..

[7] Nixon I.J., Suárez C., Simo R., Sanabria A., Angelos P., Rinaldo A., Rodrigo J.P., Kowalski L.P., Hartl D.M., Hinni M.L. (2016). The Impact of Family History on Non-Medullary Thyroid Cancer. Eur. J. Surg. Oncol..

[8] Nosé V. (2011). Familial Thyroid Cancer: A Review. Mod. Pathol..

[9] Ito Y., Miyauchi A., Oda H. (2018). Low-Risk Papillary Microcarcinoma of the Thyroid: A Review of Active Surveillance Trials. Eur. J. Surg. Oncol..

[10] Grani G., Sponziello M., Pecce V., Ramundo V., Durante C. (2020). Contemporary Thyroid Nodule Evaluation and Management. J. Clin. Endocrinol. Metab..

[11] Lamartina L., Grani G., Biffoni M., Giacomelli L., Costante G., Lupo S., Maranghi M., Plasmati K., Sponziello M., Trulli F. (2016). Risk Stratification of Neck Lesions Detected Sonographically During the Follow-Up of Differentiated Thyroid Cancer. J. Clin. Endocrinol. Metab..

[12] Ramundo V., Di Gioia C.R.T., Falcone R., Lamartina L., Biffoni M., Giacomelli L., Filetti S., Durante C., Grani G. (2020). Diagnostic Performance of Neck Ultrasonography in the Preoperative Evaluation for Extrathyroidal Extension of Suspicious Thyroid Nodules. World J. Surg..

[13] Lamartina L., Bidault S., Hadoux J., Guerlain J., Girard E., Breuskin I., Attard M., Suciu V., Baudin E., Al Ghuzlan A. (2021). Can Preoperative Ultrasound Predict Extrathyroidal Extension of Differentiated Thyroid Cancer?. Eur. J. Endocrinol..

[14] Nardi F., Basolo F., Crescenzi A., Fadda G., Frasoldati A., Orlandi F., Palombini L., Papini E., Zini M., Pontecorvi A. (2014). Italian Consensus for the Classification and Reporting of Thyroid Cytology. J. Endocrinol. Investig..

[15] Baloch Z., LiVolsi V.A. (2020). The Bethesda System for Reporting Thyroid Cytology (TBSRTC): From Look-Backs to Look-Ahead. Diagn. Cytopathol..

[16] Lamartina L., Grani G., Durante C., Filetti S. (2018). Recent Advances in Managing Differentiated Thyroid Cancer. F1000Research.

[17] Bibbins-Domingo K., Grossman D.C., Curry S.J., Barry M.J., Davidson K.W., Doubeni C.A., Epling J.W., Kemper A.R., Krist A.H., US Preventive Services Task Force (2017). Screening for Thyroid Cancer: US Preventive Services Task Force Recommendation Statement. JAMA.

[18] Lin J.S., Bowles E.J.A., Williams S.B., Morrison C.C. (2017). Screening for Thyroid Cancer: Updated Evidence Report and Systematic Review for the US Preventive Services Task Force. JAMA.

[19] Sugitani I., Ito Y., Takeuchi D., Nakayama H., Masaki C., Shindo H., Teshima M., Horiguchi K., Yoshida Y., Kanai T. (2021). Indications and Strategy for Active Surveillance of Adult Low-Risk Papillary Thyroid Microcarcinoma: Consensus Statements from the Japan Association of Endocrine Surgery Task Force on Management for Papillary Thyroid Microcarcinoma. Thyroid.

[20] Hartl D.M., Guerlain J., Breuskin I., Hadoux J., Baudin E., Al Ghuzlan A., Terroir-Cassou-Mounat M., Lamartina L., Leboulleux S. (2020). Thyroid Lobectomy for Low to Intermediate Risk Differentiated Thyroid Cancer. Cancers.

[21] Grani G., Lamartina L., Alfò M., Ramundo V., Falcone R., Giacomelli L., Biffoni M., Filetti S., Durante C. (2021). Selective Use of Radioactive Iodine Therapy for Papillary Thyroid Cancers With Low or Lower-Intermediate Recurrence Risk. J. Clin. Endocrinol. Metab..

[22] Peiling Yang S., Bach A.M., Tuttle R.M., Fish S.A. (2015). Frequent Screening with Serial Neck Ultrasound Is More Likely to Identify False-Positive Abnormalities than Clinically Significant Disease in the Surveillance of Intermediate Risk Papillary Thyroid Cancer Patients without Suspicious Findings on Follow-up Ultrasound Evaluation. J. Clin. Endocrinol. Metab..

[23] Grani G., Ramundo V., Falcone R., Lamartina L., Montesano T., Biffoni M., Giacomelli L., Sponziello M., Verrienti A., Schlumberger M. (2019). Thyroid Cancer Patients With No Evidence of Disease: The Need for Repeat Neck Ultrasound. J. Clin. Endocrinol. Metab..

[24] Ospina N.S., Salloum R.G., Maraka S., Brito J.P. (2021). De-Implementing Low-Value Care in Endocrinology. Endocrine.

[25] Lamartina L., Grani G., Durante C., Filetti S., Cooper D.S. (2020). Screening for Differentiated Thyroid Cancer in Selected Populations. Lancet Diabetes Endocrinol..

[26] Kim Y.S., Seo M., Park S.H., Ju S.Y., Kim E.S. (2020). Should Total Thyroidectomy Be Recommended for Patients with Familial Non-Medullary Thyroid Cancer?. World J. Surg..

[27] Patel K.N., Yip L., Lubitz C.C., Grubbs E.G., Miller B.S., Shen W., Angelos P., Chen H., Doherty G.M., Fahey T.J. (2020). The American Association of Endocrine Surgeons Guidelines for the Definitive Surgical Management of Thyroid Disease in Adults. Ann. Surg..

[28] Cao J., Chen C., Chen C., Wang Q.-L., Ge M.-H. (2016). Clinicopathological Features and Prognosis of Familial Papillary Thyroid Carcinoma—A Large-Scale, Matched, Case-Control Study. Clin. Endocrinol..

[29] Lee Y.-M., Yoon J.H., Yi O., Sung T.-Y., Chung K.-W., Kim W.B., Hong S.J. (2014). Familial History of Non-Medullary Thyroid Cancer Is an Independent Prognostic Factor for Tumor Recurrence in Younger Patients with Conventional Papillary Thyroid Carcinoma: Familial History of Thyroid Cancer. J. Surg. Oncol..

[30] McDonald T.J., Driedger A.A., Garcia B.M., Van Uum S.H.M., Rachinsky I., Chevendra V., Breadner D., Feinn R., Walsh S.J., Malchoff C.D. (2011). Familial Papillary Thyroid Carcinoma: A Retrospective Analysis. J. Oncol..

[31] Muallem Kalmovich L., Jabarin B., Koren S., Or K., Marcus E., Tkacheva I., Benbassat C., Steinschneider M. (2021). Is Familial Nonmedullary Thyroid Cancer A More Aggressive Type of Thyroid Cancer?. Laryngoscope.

[32] Liu L., Oh C., Heo J.H., Park H.S., Lee K., Chang J.W., Jung S.-N., Koo B.S. (2018). Clinical Significance of Extrathyroidal Extension According to Primary Tumor Size in Papillary Thyroid Carcinoma. Eur. J. Surg. Oncol..

[33] Kim H., Kwon H., Moon B.-I. (2021). Association of Multifocality with Prognosis of Papillary Thyroid Carcinoma: A Systematic Review and Meta-Analysis. JAMA Otolaryngol. Head Neck Surg..

[34] Chung S.R., Baek J.H., Choi Y.J., Sung T.-Y., Song D.E., Kim T.Y., Lee J.H. (2020). Sonographic Assessment of the Extent of Extrathyroidal Extension in Thyroid Cancer. Korean J. Radiol..

[35] Kuo E.J., Thi W.J., Zheng F., Zanocco K.A., Livhits M.J., Yeh M.W. (2017). Individualizing Surgery in Papillary Thyroid Carcinoma Based on a Detailed Sonographic Assessment of Extrathyroidal Extension. Thyroid.

[36] Hu S., Zhang H., Sun Z., Ge Y., Li J., Yu C., Deng Z., Dou W., Wang X. (2020). Preoperative Assessment of Extrathyroidal Extension of Papillary Thyroid Carcinomas by Ultrasound and Magnetic Resonance Imaging: A Comparative Study. Radiol. Med..

[37] Leong D., Ng K., Nguyen H., Ryan S. (2021). Preoperative Ultrasound Characteristics in Determining the Likelihood of Cytologically Confirmed (Bethesda VI), 1-4 Cm Papillary Thyroid Tumours Requiring Completion Thyroidectomy. Asian J. Surg..

[38] Bortz M.D., Kuchta K., Winchester D.J., Prinz R.A., Moo-Young T.A. (2021). Extrathyroidal Extension Predicts Negative Clinical Outcomes in Papillary Thyroid Cancer. Surgery.

